# *Lutzomyia* Sand Fly Diversity and Rates of Infection by *Wolbachia* and an Exotic *Leishmania* Species on Barro Colorado Island, Panama

**DOI:** 10.1371/journal.pntd.0000627

**Published:** 2010-03-09

**Authors:** Jorge Azpurua, Dianne De La Cruz, Anayansi Valderama, Donald Windsor

**Affiliations:** 1 Smithsonian Tropical Research Institute, Panamá, República de Panamá; 2 Instituto Conmemorativo Gorgas de Estúdios para la Salud, Panamá, República de Panamá; National Institute of Allergy and Infectious Diseases, United States of America

## Abstract

**Background:**

Sand flies (Diptera, Psychodidae, Phlebotominae) in the genus *Lutzomyia* are the predominant vectors of the protozoan disease leishmaniasis in the New World. Within the watershed of the Panama Canal, the cutaneous form of leishmaniasis is a continuous health threat for residents, tourists and members of an international research community. Here we report the results of screening a tropical forest assemblage of sand fly species for infection by both *Leishmania* and a microbe that can potentially serve in vector population control, the cytoplasmically transmitted rickettsia, *Wolbachia pipientis*. Knowing accurately which *Lutzomyia* species are present, what their evolutionary relationships are, and how they are infected by strains of both *Leishmania* and *Wolbachia* is of critical value for building strategies to mitigate the impact of this disease in humans.

**Methodology and Findings:**

We collected, sorted and then used DNA sequences to determine the diversity and probable phylogenetic relationships of the Phlebotominae occurring in the understory of Barro Colorado Island in the Republic of Panama. Sequence from CO1, the DNA barcoding gene, supported 18 morphology-based species determinations while revealing the presence of two possible “cryptic” species, one (*Lu.* sp. nr *vespertilionis*) within the *Vespertilionis* group, the other (*Lu. gomezi*) within the Lutzomyia-cruciata series. Using ITS-1 and “minicircle” primers we detected *Leishmania* DNA in 43.3% of *Lu. trapidoi*, 26.3% of *Lu. gomezi* individuals and in 0% of the other 18 sand fly species. Identical ITS-1 sequence was obtained from the *Leishmania* infecting *Lu. trapidoi* and *Lu. gomezi*, sequence which was 93% similar to *Leishmania (viannia) naiffi* in GenBank, a species previously unknown in Panama, but recognized as a type of cutaneous leishmaniasis vectored broadly across northern and central South America. Distinct strains of the intracellular bacterium *Wolbachia* were detected in three of 20 sand fly species, including *Lu. trapidoi*, in which it frequently co-occurred with *Leishmania*.

**Conclusions:**

Both morphological and molecular methods were used to examine an assemblage of 20 sand fly species occurring in the forests of the Panama Canal area. Two of these species, members of separate clades, were found to carry *Leishmania* at high frequency and hence are likely vectors of leishmaniasis to humans or other mammal species. A single *Leishmania* species, identified with high confidence as *Le. naiffi*, was carried by both species. That *Le. naiffi* is known to cause cutaneous lesions in South America but has hitherto not been reported or implicated in Panama opens the possibility that its range has recently expanded to include the Isthmus or that it occurs as a recent introduction. The occurrence of *Leishmania* and *Wolbachia* in *Lu. trapidoi* identifies one important vector of the disease as a potential target for gene introductions using *Wolbachia* population sweeps.

## Author Summary

Certain sand fly species living inside or on the edge of tropical forests are well known to transmit a protozoan to humans, which in lowland Panama develops into a cutaneous form of leishmaniasis; open, itching sores on the face and extremities requiring aggressive treatment with antimonial compounds. Morphological characters and DNA sequence from mitochondrial and nuclear gene fragments permitted us to identify and then establish historical relationships among 20 common sand fly species occurring in the understory of Barro Colorado Island, a forested preserve in the middle of the Panama Canal. Individuals in three of these sand fly species were found to be 26–43% infected by *Leishmania naiffi*, a species hitherto known only from the Amazonian region and the Caribbean. We then screened the same 20 sand fly species for the cytoplasmically transmitted bacteria *Wolbachia pipientis*, finding three infected at high rates, each by a distinct strain. *Lutzomyia trapidoi*, the most likely transmitter of *Leishmania* to humans in Panama, was among the *Wolbachia*-infected species, thus marking it as a possible high-value target for future biocontrol studies using the bacteria either to induce mating incompatabilities or to drive selected genes into the population.

## Introduction

New World sand flies of the genus *Lutzomyia* are exclusively responsible for transmitting cutaneous and visceral leishmaniasis (CL and VL) to humans, diseases caused by kinetoplast protozoans of the genus *Leishmania* [1]. While the Isthmus of Panama is a region crucial to world commerce, growing tourism and increasing human population, it also remains an area where leishmaniasis continues to be endemic. Below, we report the results of a study using molecular techniques to clarify species relationships and *Leishmania* infection status of common sand fly species occurring in the understory of the mature tropical forest on Barro Colorado Island, located in the central watershed of the Panama Canal. The study additionally tests sand fly species for infection by th rickettsia, *Wolbachia pipientis*, a microbe which could prove useful in future efforts to control species which vector the disease to man.

*Leishmania* infection is characterized by a species-specific pathology, varying from cutaneous lesions to the potentially fatal visceral form. Found primarily in tropical latitudes, 12 species regularly infect humans in the Neotropics, whereas only 6 species are reported to do so in the Paleotropics [2]. The global burden of the disease has been estimated to be about 500,000 cases of visceral leishmaniasis and 1.1–1.5 million for cutaneous leishmaniasis (CL) per year [3],[4]. The parasites are transmitted as metacyclic promastigotes, delivered by the bite of phlebotomine sand flies. Once in the human host, they become intracellular amastigotes that spread through binary fission causing symptoms through cell lysis [1]. Humans and a considerable number of other mammals can act as reservoirs for the parasite. There has been a noted world-wide increase in the resistance of *Leishmania* to traditional pentavalent antimonial treatments and the response rate for alternate treatments is species-specific [1],[5] raising the importance of accurate species diagnosis. Some strains of *Leishmania* are spreading northward, with cases reported in previously non-endemic areas of North Texas [6].

Some *Lutzomyia* sand fly species are infected with the alpha-proteobacterium (*Rickettsia*) *Wolbachia pipientis* [7], a reproductive parasite of insects [8]–[11]. *Wolbachia* infects a wide variety of arthropod phyla (including insects, crustaceans and arachnids) and is an endosymbiont within filarial nematodes [12]–[14]. *Wolbachia* cells concentrate in the reproductive tissues and are transmitted vertically to offspring through egg cytoplasm and horizontally between species through unknown mechanisms. *Wolbachia* is known to infect at least 15–20% of insects worldwide [15],[16], and in most filarial nematode species infecting humans [17]. *Wolbachia* manipulates host reproduction in diverse ways, such as male-killing, feminization of genetic males [18], parthenogenesis induction [19], and by causing cytoplasmic incompatibility (CI) of host gametes [20]–[22]. These adaptations benefit the bacterium by allowing it and the mitochondrial genome of its host to spread quickly through uninfected populations [23],[24]. Because of the sweep, *Wolbachia* has been explored as a potential driver for introducing transgenes into natural populations [25]–[27]. Low levels of maternal transmission in some phlebotomine species may hamper attempts to use it in transgenic strategies [28] but to date this has not been investigated in New World sand flies. However, if *Wolbachia* could be used as a population control mechanism analogous to sterile male release, then less than perfect transmission actually may be desirable [29].

Identification of *Lutzomyia* sand flies has traditionally been based on morphological characters found in the terminal segments of the abdomen. Morphology-based classification is sometimes weakened by intra-species variation, the occurrence of cryptic species [30], and the lack of distinguishing morphological characters in the females of some species (ie. the *Vespertilionis* Group) [31]. Thus, for phlebotomine sand flies, DNA sequencing provides a useful independent assessment of species relationships. In particular, the cytochrome oxidase 1 mitochondrial gene (CO1) has been used for this purpose in many arthropods [32] and is employed extensively by the Barcode of Life Initiative. This gene has been successfully used to distinguish species [33], and to discover cryptic species in multiple insect orders [34],[35], however, its utility as a tool in phylogenetic reconstruction remains controversial [36] unless complemented by sequence from the nuclear genome. Accurate identification of *Lutzomyia* species and species groups will be important in establishing baselines for understanding the spread of the disease and eventually in targeting pest control measures to mitigate *Leishmania* transmission.

Previous attempts at constructing a phylogeny of *Lutzomyia* flies have focused on ribosomal genes [31]. The 18S rDNA gene corresponds to RNA transcripts in the small (40S) ribosomal subunit, containing highly conserved nucleotide sequence, as well as highly variable loops and has been used to elucidate phylogenetic relationships for diverse taxa, including *Lutzomyia* [37]. We used two fragments of the 18S nuclear gene extracted from individual sand flies to reconstruct the phylogenetic relationships between the common *Lutzomyia* species found on Barro Colorado Island, Panama. Separate original infection events can be reasonably inferred if *Leishmania* infects species occurring in separate, well-supported clades.

## Materials and Methods

### Phlebotomine Survey

**Figure 1:**
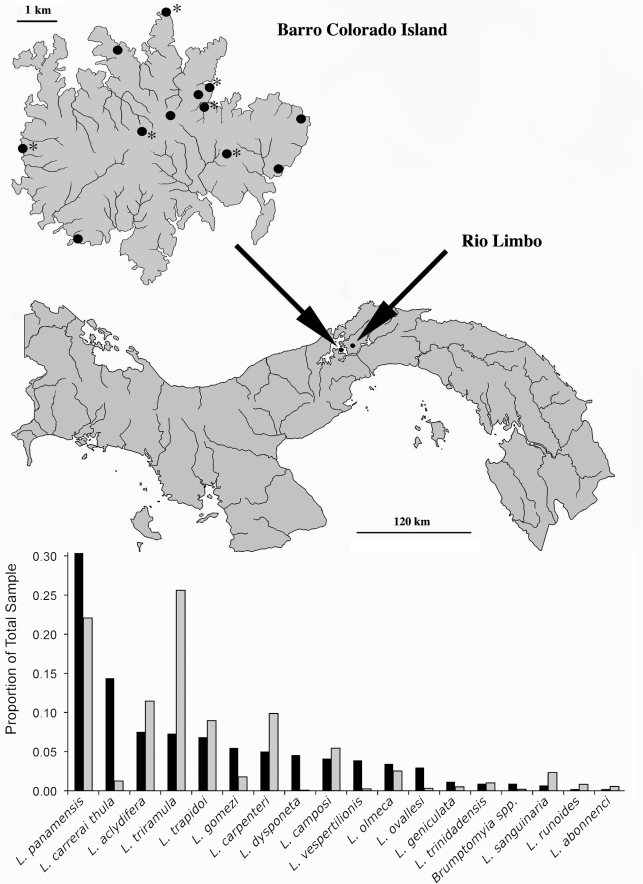
Collecting sites on Barro Colorado Island (above) with asterisks indicating sites from which *Leishmania* positive sand flies were detected and the frequencies of each of the Phlebotominae species collected in this study (below; black bars) compared to those recorded by Chaniotis et al. (1971) at Rio Limbo on the mainland (gray bars), approximately 10 km to the East.

Sand flies were collected on thirteen different dates at one to two week intervals between May 12, 2007 and September 12, 2007 at sampling sites distributed across Barro Colorado Island (Fig. 1). At each site, four “CDC” light traps [38], were placed at 25m intervals in the understory for 12 hours (6PM to 6AM). Each trap was modified to accommodate a small LED light to increase the catch and a 50ml plastic cup half filled with 95% ethanol to rapidly kill and fix small insects. The catch was removed from the traps at the end of each trapping period and stored at −20°C. Four additional specimens were removed from CDC traps run at km 1 of the “Pipeline Road” N of Gamboa, and from malaise trap samples taken near the bases of the canopy cranes at San Lorenzo and Parque Nacional Metropolitano during the same time period (2). Male and female sand flies were identified by a highly experienced *Lutzomyia* taxonomist (R. Rojas) using the keys to species groups [39].

### DNA Extraction

DNA extractions were carried out using QIAGEN Puregene Gentra alcohol and salt precipitation. Sand flies were frozen in liquid nitrogen, crushed and run through the kit's default protocol. As the DNA pellet was very small, the final ethanol wash was removed via micropipette.

Single *Lutzomyia* and *Brumptomyia* extracts were used as templates for PCR amplification. We targeted a conserved region of the *Leishmania* minicircle DNA (a non-nuclear sequence in the kinetoplast organelle present only in protozoans of the class Kinetoplastea) that is copied many-fold and contains a conserved region suitable for PCR with primers 13A and 13B [40]. This approach has been shown to be sensitive and reliable [41]. Confirmation of *Leishmania* infection was attempted with other primer sets such as 13Y+13Z [40] and LSUC+LSUL [42] but spurious amplification was seen, possibly from non-specific annealing to sand fly or sand fly blood meal DNA [43] (for discussion on non-specific amplification). Confirmation of infection was easily achieved using primers L5.8S+LITSRn [41] to amplify the ITS-1 region on the *Leishmania* nuclear genome.

### Polymerase Chain Reaction (PCR)

A 20 µL PCR mix consisted of a final concentration of 1× buffer, 1.25mM MgCl_2_, 0.2 mM dNTP, 1 µM of each of the two primers, and 0.5 units of Taq polymerase (Applied Biosystems AmpliTaq). The thermal cycler protocol depended on the gene being amplified.

#### *Lutzomyia* cytochrome oxydase 1

We used universal CO1 primers [32]. Thermal cycle protocol: initial melting 94°C for 2 minutes; 37 cycles of melting at 94°C for 30 seconds, annealing at 55°C for 45 seconds, extension at 72°C for 1 minute 30 seconds. A final extension time of 10 minutes at 72°C was followed by holding at 4°C.

#### *Lutzomyia* 18S

Primers were 18SA F&R and 18SB F&R [44]. Thermal cycler protocol included a ‘touch-up’ step: initial melting 94°C for 2 minutes; 10 cycles of melting at 94°C for 35 seconds, annealing at 51.4°C for 30 seconds, extension at 72°C for 30 seconds; 24 cycles of melting at 94°C for 30 seconds, annealing at 54.8°C for 30 seconds, and extension at 72°C for 40 seconds. A final extension time of 10 minutes at 72°C was followed by holding at 4°C.

#### *Leishmania* kinetoplast

Primers were 13A and 13B [40]. Thermal cycler protocol as CO1, except annealing was 60°C.

#### *Leishmania* ITS-1

Primers were ITS-1 primers L5.8s and LITSRn, general to all *Leishmania* [45].Thermal cycle protocol: initial melting 95°C for 4 minutes; then 36 cycles of melting at 95°C for 40 seconds, annealing at 58°C for 30 seconds, and extension at 72°C for 1 minute. A final extension time of 6 minutes at 72°C was followed by holding at 4°C.

#### *Wolbachia* 16S

Primers were WSPEC specific to *Wolbachia* 16S [16]. Thermal cycler protocols were as in CO1, including annealing temperature.

#### *Wolbachia* MLST genes

Primers are degenerate MLST primers [46]. Thermal cycler protocols were as in CO1 except annealing temperatures were 54°C for coxA, ftsZ, gatB, hcpA and 59°C for WSP and fbpA.

### DNA Sequencing

Leftover primers and dNTPs were removed from PCR products via simultaneous incubation with exonuclease and shrimp alkaline phosphatase (ExoSAP, USB Corporation). For some PCR reactions, non-specific products were visible, so the desired product band was cut followed by Gelase (Epicentre Biotechnologies) digestion. Purified PCR products were then sequenced by BigDye Terminator 3.1 cycle sequencing kit (Applied Biosystems). Sequencing reactions were cleaned using Sephadex G-50 columns on Millipore Multiscreen 96-well filtration plates or via BigDye Xterminator kit (Applied Biosystems). The clean sequencing reaction was run through an ABI 3130× sequencer.

### Phylogenetic Tree Construction

Sequences were examined using Sequencher 4.7 (Gene Codes Corporation, 2006). Ends were trimmed to clearly defined peaks. Forward and Reverse sequences for each sample were compared to correct dye blots and to ensure non-contamination. Sequences were aligned with Clustal W [47] online implementation [48], and trimmed to equal length before analysis in PAUP 4.0 [49], ModelTest [50], and MrBayes [51],[52].

The phylogenetic relationships among *Lutzomyia* species were investigated by sequencing two fragments of the 18S gene (18SA 402bp, 18SB 482bp) from single *Lutzomyia* extracts. The two sequences were aligned separately using Clustal W2 [47],[48]; then the concatenated sequence was analyzed using ModelTest for AIC and hLTR best-fit models of evolution [50]. The hLTR recommendation by ModelTest was a General Time Reversible model with invariant site (I) and gamma shape (G) parameter adjustments. The AIC recommendation was the Hasegawa-Kishino-Yano 1985 (HKN85) model, with I and G adjustments. These models were implemented in the Maximum Likelihood analysis in PAUP for tree construction, and gave identical topologies. The ML tree was bootstrapped using the HKN85 model. The concatenated sequences were also used for Bayesian Inference (BI) analysis with MrBayes [51],[52]. The HKN85 model was implemented in MrBayes so that BI posterior probabilities could be directly compared to bootstrap values in ML from PAUP. All trees were generated using *Brumptomyia galindoi* as an outgroup.

Highly variable loop regions in 18S where alignment was difficult or non-existent were excised after alignment. No attempt was made to code gaps as binary or multi-state characters.

CO1 barcode sequence was obtained from 49 individuals identified by morphology in 18 species. More than one specimen from each species was sequenced when available (Table 1). We aligned sequences manually as no insertions or deletions were present in the protein-coding fragment. Sequence reliability was determined by comparing the forward and reverse reads, as well as *in silico* translation using the invertebrate mitochondria genetic code to ensure nonsense codons were not present in the fragment. Aligned sequences were exported to PAUP* 4.0 [49] where a neighbor-joining tree was generated using a Kimura 2-Parameter correction for DNA distances.

**Table 1:** Species list and abundance of Phlebotominae recovered from the understory on Barro Colorado Island, Panama.

Species	*Subgenus (Group) - Series*	Individuals Collected	% (N) Individuals Infected		Genbank Accession Numbers		
			*Leishmania*	*Wolbachia*	COI	18Sa	18Sb
*Lutzomyia carpenteri* (Fairchild and Hertig)	*(Aragaoi) - aragaoi*	22	0 (20)	0 (20)	GU001729-31	FJ977587	GU048921
*Lutzomyia runoides* (Fairchild and Hertig)	*(Aragaoi) - brasiliensis*	1	0 (1)	0 (1)	GU001754	FJ977598	GU048922
*Lutzomyia aclydifera* (Fairchild and Hertig)	*(Dreisbachi)*	33	0 (20)	0 (20)	GU001724, 25	FJ977588	GU048918
*Lutzomyia triramula* (Fairchild and Hertig)	*(Longispina)*	32	0 (22)	0 (20)	GU001766, 67	FJ977589	GU048911
*Lutzomyia trinidadensis* (Neustead)	*(Oswaldoi)*	4	0 (2)	0 (4)	GU001765	FJ977593	GU048926
*Lutzomyia abonnenci* (Floch and Chassignet)	*(Shannoni)*	1	0 (1)	0 (1)	—	—	—
*Lutzomyia ovallesi* (Ortiz)	*(Verrucarum) - verrucarum*	13	0 (13)	0 (13)	GU001744-46	FJ977594	GU048916
*Lutzomyia vespertilionis* (Fairchild and Hertig)	*(Vespertilionis)*	15	0 (15)	73.3 (15)	GU001768, 69, 71,72	FJ977590	GU048910
*Lutzomyia* sp. nr *vespertilionis*	*(Vespertilionis)*	1	0 (1)	100 (1)	GU001770	FJ977591	GU048909
***Lutzomyia sanguinaria*** ** (Fairchild and Hertig)**	*(Vexator)*	3	0 (3)	0 (3)	GU001755-57	FJ977592	GU048923
***Lutzomyia gomezi*** ** (Nitzulescu)**	*Lutzomyia - cruciata*	24	26.3 (19)	0 (20)	GU001737-39	FJ977599	GU048914
***Lutzomyia olmeca*** ** (Vargas and Diaz-Najera)**	*Nyssomyia*	15	0 (15)	0 (15)	GU001741-43	FJ977585	GU048919
***Lutzomyia trapidoi*** ** (Fairchild and Hertig)**	*Nyssomyia*	30	43.3 (30)	53.3 (30)	GU001758-64	FJ977595	GU048920
*Lutzomyia camposi* (Rodriguez)	*Pressatia*	18	0 (18)	0 (18)	GU001726-28	FJ977582	GU048912
*Lutzomyia dysponeta* (Fairchild and Hertig)	*Pressatia*	20	0 (18)	0 (19)	GU001732, 33	FJ977583	GU048913
***Lutzomyia carrerai thula*** ** (Young)**	*Psychodopygus - panamensis*	63	0 (18)	0 (16)	GU001751-53	FJ977584	GU048924
*Lutzomyia guyanensis ( = geniculata)* (Floch and Abonnenc)	*Psychodopygus - panamensis*	5	0 (5)	0 (5)	GU001736	FJ977586	GU048925
***Lutzomyia panamensis*** ** (Shannon)**	*Psychodopygus - panamensis*	133+	0 (29)	0 (29)	GU001747-50	FJ977596	GU048917
*Brumptomyia galindoi* (Fairchild and Hertig)		3	0 (3)	0 (3)	GU001734, 35	FJ977581	GU048908
*Brumptomyia hamata* (Fairchild and Hertig)		1	0 (1)	0 (1)	GU001740	—	—

The sand fly species collected (man-biting species in bold), their higher classification, the numbers of field-collected individuals, the percent and number which tested positive for Leishmania and Wolbachia using PCR and the Genbank accession numbers for the COI bar-code gene and two fragments of the nuclear-ribosomal 18S gene.

## Results

### Phlebotomine Collection

More than 437 sand fly individuals collected from seven shoreline and five interior forest sites on Barro Colorado Island, Panama contained 17 *Lutzomyia* and 2 *Brumptomyia* species determined by morphology (Table 1). Abundance of *Lu. panamensis*, the numerically dominant species was not accurately determined due to its extremely high numbers (thousands) in the combined sample. Less abundant species were counted precisely and ranged from one to a total of 63 individuals per species. Five man-biting species (*Lu. panamensis*, *Lu. trapidoi*, *Lu. olmeca*, *Lu. gomezi* and *Lu. carrerai thula*) accounted for more than 61% of individuals collected on BCI. The relative abundances of the species collected in this study were significantly correlated to those found in a previous intensive study of sand fly community composition [53] at the “Rio Limbo” site on the mainland, 7 km NW of Gamboa and 10 km east from BCI (r_s_ = 0.64, n = 18, p<0.01) (Fig. 1). That 16-month study collected over 30,000 *Lutzomyia* individuals in 35 species, with *Lu. triramula* and *Lu. panamensis* comprising the two most abundant species coming to understory light traps during the May to August period. A seventh man-biting species, *Lu. ylephiletrix* (Fairchild and Hertig), collected in the Rio Limbo study was not present in BCI samples.

The seasonal trends in adult population density recorded by the “Rio Limbo” study [54],[55], suggest 11 of the species collected in the present study (including three man-biting species) reach their peak densities in the early wet season, three in the late wet and three others in the dry season. Of the six man-biting species in our samples, three principally are canopy species but all can occur at different levels within the forest. Five species are nocturnal feeders with peaks in activity at dawn and dusk, while *Lu. carrerai thula* actively feeds at all hours.

### CO1 Barcoding Gene and Sand Fly Species

**Figure 2:**
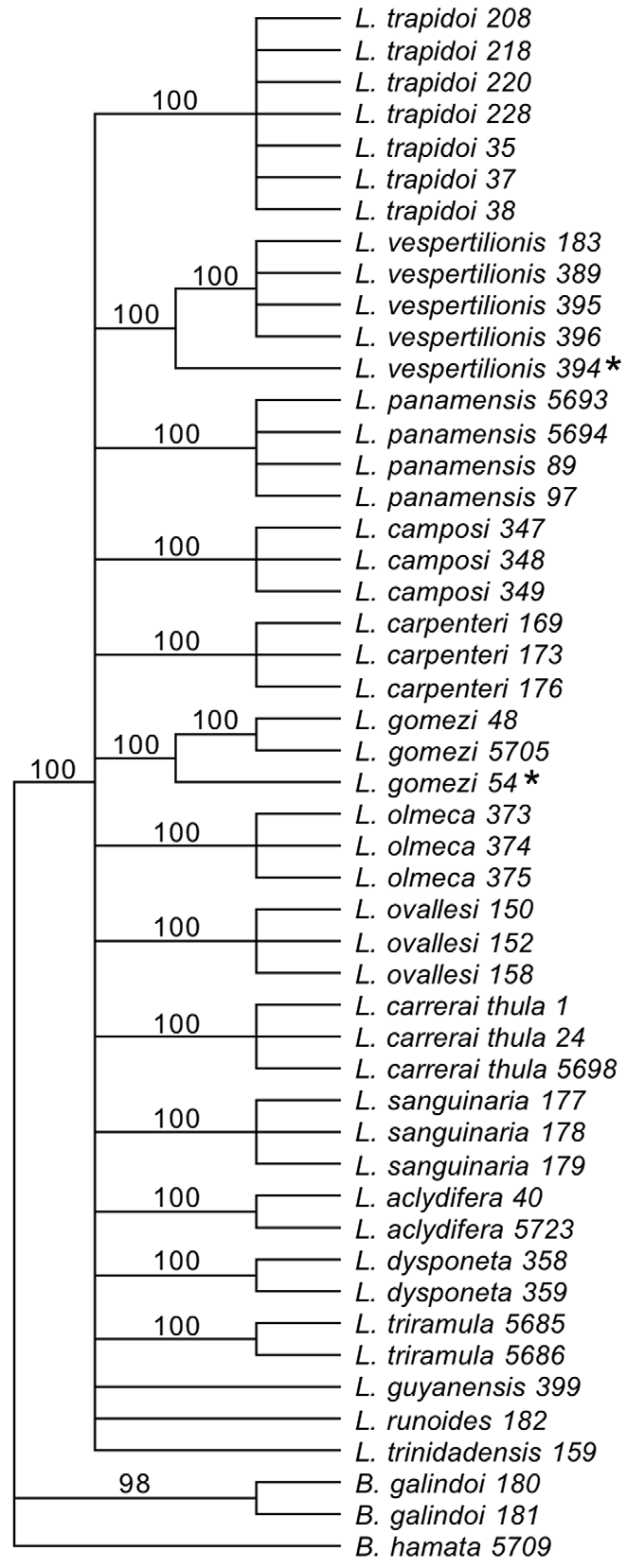
Neighbor-Joining, COI consensus tree showing only nodes supported by 100% bootstrap values based on 1000 replicates. The 49 individuals sequenced were initially identified using genitalic characters into the two “outgroup” *Brumptyomyia* species and 16 “ingroup” *Lutzomyia* species. Possible “cryptic” species indicated by asterisks are present within both the *Lu. gomezi* and *Lu. vespertilionis* nodes.

Neighbor-joining analysis of CO1 sequence extracted from 49 sand fly specimens separated the genera, Brumptomyia and Lutzomyia, and unambiguously assigned 43 individuals to13 morphological species (Fig. 2). The status of the three species represented by single specimens remained unresolved within the Lutzomyia clade but occurred separate from the 13 other morphological species. Single specimens within two morphological species groups diverged sufficiently from conspecifics to possibly indicate species differences. The separation of specimen 394 within *Lu.vespertilionis*, received 100% bootstrap support as did specimen 54 within *Lu. gomezi*. Thus, the presence of two cryptic or misdiagnosed species within our group of 16 morphologically determined *Lutzomyia* species is suggested.

### Phylogenetic Analysis using Nuclear Sequence

**Figure 3:**
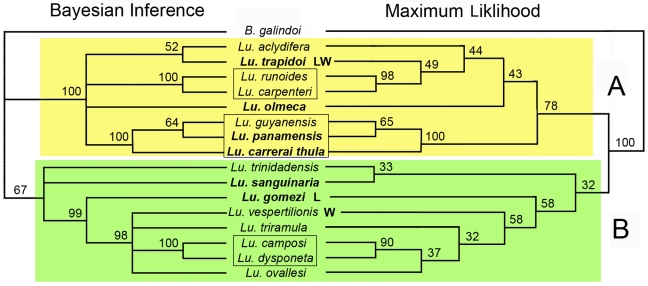
Bayesian Probability and Maximum Likelihood trees based on 500 bp of the 18s gene sequenced from 17 Phlebotominae species. The topologies produced by these approaches identify two deep clades each containing the same set of species. Man-biting species are in bold type. Infection status for *Lutzomyia* and *Wolbachia* are indicated by “L” and “W”.

Both Maximum Likelihood (ML) and Bayesian Inference (BI) trees apportioned the 16 *Lutzomyia* species identically among two apparently deep clades within the genus *Lutzomyia* (Fig. 3). Both trees recovered the three species within the Psychodopygus group as a monophyletic group within Clade “A”. Further, the two species within the Aragaoi group were recovered as a separate subgroup within Clade “A”. Within clade “B” only the Pressatia subgenus was recovered with high support in both trees. The two *Leishmania* infected species, *Lu. trapidoi* and *Lu. gomezi*, occur isolated within separate, well-supported clades in both BI and ML trees. Similarly, the two *Wolbachia* infected species, *Lu. trapidoi* and *Lu. vespertilionis*, occur in separate, well-supported clades.

### Leishmania Infections

Only *Lu. trapidoi* and *Lu. gomezi* of 20 species tested gave replicable amplification of the kinetoplast gene fragment. The prevalence of *Leishmania* ranged from 0% in 18 species to 26.3% in *Lu. gomezi* and 43.3% in *Lu. trapidoi* (Table 1). We collected one or both of these species at six of our 12 collecting sites on BCI, including traps deep within the forest and near the edge of the island.

When the amplified products from the ITS-1 region were sequenced and BLASTed against the nucleotide collection in GenBank, the closest match between *Leishmania* found in *Lu. trapidoi* and *Lu. gomezi* was found with *Le. naiffi* (100% coverage, 94% identity; **GenBank Accession Number 76577722**). The next closest match was *Le. lainsoni* (73% coverage, 96% identity). As a control, we sequenced a serum sample from blood infected with *Le. panamensis*. The amplification band and sequence were different from that obtained from infected BCI *Lutzomyia* spp. and corresponded (100% coverage, 99% identity) to *Le. panamensis* **(GenBank Accession Number 2764481)**.

*Wolbachia Infections*. Single *Lutzomyia* and *Brumptomyia* extracts were used as templates for a PCR reaction using the WSPEC *Wolbachia* specific primer [16]. Gel electrophoresis showed two species, *Lu. trapidoi* and *Lu. vespertilionis*, were infected. The prevalence of *Wolbachia* ranged from 0% in 17 species to 53.3% in *Lu. trapidoi*, and 68.8% in *Lu. vespertilionis*. The species, *Lu.* sp. nr *vespertilionis*, also tested positive for *Wolbachia* (Table 1).

*Wolbachia* multi-locus sequence typing (MLST) is preferred over the surface-protein (WSP) for characterizing Wolbachia strains [46]. We amplified fragments of five MLST genes and the WSP gene [56] for four individuals from each of the sand fly species groups testing positive for *Wolbachia*. We found that *Lu. trapidoi* individuals were uniformly infected with a single *Wolbachia* strain. Within the *Vespertilionis* species group three specimens were infected by a single *Wolbachia* strain, while the fourth specimen with the divergent CO1 sequence (no. 394, *Lu.* sp. nr *vespertilionis*) carried a second *Wolbachia* strain (Table 2).

**Table 2:** Multilocus strain-types (MLST) of *Wolbachia* extracted from infected *Lutzomyia* species.

Species	Individual ID	WSP	fbpA	coxA	ftsZ	gatB	hcpA
*Lu. trapidoi*	35	5	26	2	21	23	hcpA-a
	208	5	26	2	21	23	hcpA-a
	218	5	26	2	21	23	hcpA-a
	220	5	26	2	21	23	hcpA-a
*Lu. vespertilionis*	183	wsp-a	fbpA-a	66	ftsZ-a	gatB-a	hcpA-b
	389	wsp-a	fbpA-a	66	ftsZ-a	gatB-a	hcpA-b
	396	wsp-a	fbpA-a	66	ftsZ-a	gatB-a	hcpA-b
*Lu. sp. nr vespertilionis*	394	wsp-b	26	coxA-a	21	23	86

a Numerical responses within cells identify alleles occurring in the general Wolbachia MLST data base [56] whose sequences are identical to those recovered in this study. Non-numeric entries indicate allele sequences unique to this study.

From the 20 species tested for both *Wolbachia* and *Leishmania* infections, two species (*Lu. vespertilionis* and *Lu.* sp. nr *vespertilionis*) were solely infected by *Wolbachia*, one species (*Lu. gomezi*) was solely infected by *Leishmania*, and one species (*Lu. trapidoi*) was infected by both *Wolbachia* and *Leishmania*. Among the 30 *Lu. trapidoi* individuals tested for both types of infection, 13 individuals (43.3%) were positive for *Leishmania* and 16 individuals (53.3%) were positive for *Wolbachia*. The seven double infected individuals were no more common than would be expected by chance (Fisher's exact test, p = 0.99, n = 30).

## Discussion

Neighbor-joining analysis clustered the CO1 sequences obtained from 46 individuals into 13 taxa previously determined by spermathecal morphology. However, one specimen was discovered within each of two species groups (the subgenus and series, *Lutzomyia-cruciata* and the group, *Vespertilionis*) containing sequence sufficiently divergent from conspecifics to identify them as likely cryptic or misdiagnosed species. Sequencing larger numbers of individuals might increase the numbers of individuals with divergent mitochondrial haplotypes. The utility of CO1 sequencing as a tool for detecting species level genetic variation within morphologically determined species of New World Phlebotominae seems substantial. However, while analyses of CO1 sequence give high levels of support for species groups, more basal nodes were unsupported regardless of method of tree construction. Homoplasies, mitochondrial sweeps caused by *Wolbachia* [57] or genetic drift likely limit the usefulness of CO1 for phylogeny reconstruction in *Lutzomyia*, a conclusion reached in other studies [31].

Individual no. 394 in the *Vespertilionis* group with aberrant CO1 was sequenced only after we discovered it contained a different *Wolbachia* strain MLST (see below). The females of both *Lu. vespertilionis* and *Lu. isovespertilionis* (Fairchild & Hertig), are practically indistinguishable. Further they are the only two species in this group known to occur in Panama. Only subtle difference in the male paramere allow the two species to be determined [39]. Alternatively, individual no. 394 may not be *Lu. isovespertilionis* but an undescribed, cryptic species. This ambiguity will likely remain until additional collecting and sequencing of the *Lu. vespertilionis* species group is carried out. Below we provisionally refer to this individual as *Lu.* sp. nr *vespertilionis*.

Our attempt to recover phylogenetic relationships among Panamanian sand flies is based on two fragments of the nuclear ribosomal 18S gene (Fig. 3). While the information within this relatively short (884 bp) segment limits the support for certain nodes within the tree, the separation of all taxa into either the A or B clades is well supported by both Bayesian Inference and Maximum Likelihood analysis. There is bootstrap support at three nodes within the tree representing *Pressatia* (Mangabeira) and *Psychodoygus* subgenera and the *Aragoi* group (Theodor).

*Leishmania* infection was confirmed by PCR in two of the 18 *Lutzomyia* species collected on BCI. Both are man-biting species with high rates of infection, 43.3% and 26.3%, in *Lu. trapidoi* and *Lu. gomezi*, respectively. *Leishmania* has been discovered previously in both of these species in Panama as well as in *Lu. ylephiletrix* and *Lu. panamensis* [58]. We found none of 29 individuals of the numerically dominant species, *Lu. panamensis*, infected in our study. However, the rate of infection in *Lu. panamensis* reported by Christensen et al. [58] was extremely low (1.2%; 4 of 306) and accordingly, we may easily have missed infected individuals by chance.

Methods for detection, diagnosis and species identification of *Leishmania* parasites currently in use are microscopy of clinical samples, PCR, and RFLP, each with a different degree of sensitivity, rate of false negatives and positives, and species-specificity. We successfully detected *Leishmania* from single sand fly DNA extracts with a single PCR step using the general kinetoplast primers and general ITS-1 primers. Previous work has also shown this is possible with semi-nested PCR [37] and with genus-specific primers [59]. Other work has shown that PCR amplification is as reliable as visual identification when applied to lab-infected strains [60]. Direct comparisons of the reliability of our approach are not possible as we did not try to detect the parasites any other way, such as by visual inspection of collected *Lutzomyia* flies. However, it is noteworthy that two primer sets developed under different circumstances for different purposes yielded congruent results.

The detection of *Leishmania (Viannia) naiffi* in *Lu. trapidoi* and *Lu. gomezi* is, to our knowledge, the first record of this species in Panama. Both sand fly species are known carriers of *Leishmania panamensis*, with *Lu. trapidoi* considered the main vector of CL in Panama (Panamerican Health Organization Bulletin 44, 1997). *Leishmania naiffi* is known to occur widely in South America and the Caribbean, including Brazil, Ecuador, Peru, French Guiana, and Martinique where it causes CL in humans [61]. The presence of *Le. naiffi* has been confirmed in tissue samples of humans in the Amazon region (Panamerican Health Organization Bulletin 44, 1997). The main reservoir for the parasite is thought to be the armadillo *Dasypus novemcinctus* [62], a mammal distributed widely across the Americas including Barro Colorado Island and mainland Panama.

There are several hypotheses that would account for the presence and late discovery of *Le. naiffi* in Panama: 1) *Leishmania naiffi* occurrs naturally in Panama but early surveys of *Leishmania* overlooked or misidentified it because of limitations in methodology, 2) the primarily South American range of *Le. naiffi* has expanded northward to include the Isthmus in the past 40 years, perhaps because of climate change or deforestation (Barro Colorado Island has a clear record of increasing night time temperature, a factor that certainly could affect the nocturnal and crepuscular activity of sand flies.), 3) *Leishmania naiffi* was introduced to the Caribbean coast and canal area by workers coming to Panama from the West Indies to construct the Panama Railroad and/or the Panama Canal during the mid to late 1800's and the first two decades of the 1900's, or 4) *Le. naiffi* was accidentally introduced to BCI during the late 1960's by the import of exotic primate species collected by ethologists in the upper Amazon drainage. Further molecular analysis directed toward *Leishmania* vectoring sand fly species (especially *L. trapidoi* and *L. gomezi*) collected on the adjacent mainland and sites in the Darien Province should provide an easy test of these hypotheses. Indeed, much stands to be gained by a geographic survey of sand flies across the entire Isthmus using molecular methods of detection and sequence comparison.

*Wolbachia* was initially detected in 2 of 17 sand fly species surveyed (11.7%). This is lower than reported by a previous study in the Paleotropics (27%) [9] and estimates for arthropod species globally (16–20%) [29]. MLST analysis reveals that individuals in the *Vespertilionis* group are separately infected by two different strains of *Wolbachia*, with no indication of simultaneous infection. Three *Lu. vespertilionis* specimens carried one *Wolbachia* strain, while a divergent strain was carried by a fourth specimen. We confirmed the specimen with the divergent strain was genetically distinct from other *Lu. vespertilionis* flies (Fig. 1) by comparing CO1 sequences from the four specimens. Effectively, this results in 3 out of 18 (16.6%) *Lutzomyia* species positive for *Wolbachia*. We speculate that *Wolbachia* may be part of a genetic barrier that separates the very similar species, *Lu. vespertilionis* and *Lu.* sp. nr *vespertilionis*, but more research is required to confirm the species identities and the occurrence of intra-specific *Wolbachia* incompatibilities. The extent to which *Wolbachia* induced cytoplasmic incompatibility contributes to species divergence in sand flies [63] deserves additional study.

MLST shows that the *Wolbachia* strains carried by *Lu. vespertilionis* and *Lu.* sp. nr *vespertilionis* are highly divergent and hence may represent independent acquisitions by horizontal transfer. It is important to note that the *Wolbachia* strain infecting *Lu.* sp. nr *vespertilionis* shares three of the six alleles found in the *Lu. trapidoi Wolbachia* strain, while sharing no alleles with the *Wolbachia* stain in *Lu. vespertilionis*. This may indicate that the agent responsible for stochastic horizontal transmission of *Wolbachia* occurs in or interacts with both *Lu. trapidoi* and the cryptic species. The considerable phylogenetic distance between the species in the *Vespertilionis* group and *Lu. trapidoi* present in the 18S tree (Fig. 3) suggests that the *Wolbachia* strains carried by these two species are unlikely to have descended from a single transfer event.

The moderate *Wolbachia* infection rate we recorded within sand fly species (Table 1) is a feature consistent with other *Wolbachia* research. This was also seen in studies of *Phlebotomus papatasi*, and was due to low vertical maternal transmission [28]. If low maternal vertical transmission also occurs in New World phlebotomines, then *Wolbachia* will probably not be useful as a mechanism for driving transgenes into sand fly populations, as has been studied with mosquitoes [64]. If *Wolbachia* were to be used in a manner analogous to sterile males, low transmission may actually increase the effectiveness due to the population staying polymorphic with *Wolbachia* infection [29]. *Wolbachia* is possibly already limiting *Lu. trapidoi* numbers naturally. It is also important to note that while *Wolbachia* produces CI in Old World sand flies, its phenotype in New World *Lutzomyia* species remains unknown.

Our goal after testing species boundaries within *Lutzomyia* using the CO1 barcoding gene, was to examine phylogenetic relationships of the sand fly species in our samples. We found that the two 18S fragments we sequenced contain useful information and amplified with minor optimization. However, greater resolution ultimately will be needed, perhaps by combining sequence from 18S and 28S [31]. Nevertheless, the phylogeny we obtained based solely on 18S clearly indicates the two sand fly vectors in this study and a third species known to vector *Le. panamensis* (*Lutzomyia panamensis*) are not closely related, indicating their vectoring capacities may have evolved independently. Although no clinical data is available indicating *Le. naiffi* infects humans in Panama, residents of BCI and surrounding mainland areas have developed *Leishmania* symptoms and received antimonial treatment. The occurrence of *Wolbachia* in one of the principal vectors of leishmaniasis may provide an extra tool for future control of this disease on the Isthmus and perhaps elsewhere in the Neotropics.

## Supporting Information

Alternative Language Abstract S1 Spanish translation of the abstract by JA.
(0.03 MB DOC)
