# Supplementary material for: Lutzomyia Sand Fly Diversity and Rates of Infection by Wolbachia and an Exotic Leishmania Species on Barro Colorado Island, Panama
Source: PLoS Negl Trop Dis. 2010 Mar 9;4(3):e627. doi: 10.1371/journal.pntd.0000627 (PMC2834748; doi:10.1371/journal.pntd.0000627)
Supplement: Alternative Language Abstract S1 — Spanish translation of the abstract by JA. (0.03 MB DOC) [file pntd.0000627.s001.doc]

**Resumen**

**Contexto**

Los mosquitos flebótomos (Diptera, Psychodidae, Phlebotominae) del género *Lutzomyia* son los transmisores predominantes en el Nuevo Mundo de la leishmaniasis, una enfermedad protozoaria. Dentro del estuario del Canal de Panama, la forma cutánea de la leishmaniasis es una amenaza continua para los residentes, turistas, y miembros de la comunidad internacional de investigación científica. Aquí reportamos los resultados de exámenes para detectar la presencia de *Leishmania* en un ensamblaje de mosquitos de bosque tropical, y para detectar un microbio que potencialmente puede servir para el control de transmisión (*Wolbachia pipientis*, una rickettsia transmitida citoplasmáticamente). Saber con precisión cuáles especies de *Lutzomyia* están presentes, cuáles son sus relaciones evolucionarías y con cuáles cepas de *Leishmania* y *Wolbachia* están infectadas es de valor critico para construir estrategias para mitigar el impacto de esta enfermedad en los seres humanos.

**Metodología y Resultados**

Colectamos, organizamos, y secuenciamos el ADN de los mosquitos flebótomos para determinar el nivel de diversidad y las relaciones filogenéticas que ocurren en la capa subyacente de vegetación de la isla Barro Colorado en la República de Panamá. Secuencia de CO1, el gen de código de barra, apoyo 18 determinaciones de especia basadas en morfología, además de revelar la presencia de dos posibles especies ‘cripticas’, una (*Lu. sp. nr vespertilionis*) dentro del grupo *Vespertilionis*, y la otra (*Lu. Gomezi*) dentro de la serie Lutzomyia-cruciata. Usando cebadores para la región ITS-1 y el ‘minicirculo’ cinetoplasto detectamos ADN de *Leishmania* en 36.7% de *Lu. trapidoi*, y 26.3% de *Lu. gomezi*, y 0% en 18 otras especies de mosquito flebótomo. Las secuencias de ITS-1 obtenidas de *Lu. trapidoi* y *Lu. gomezi* fueron idénticas, y con una similitud de 93% a la secuencia de *Leishmania (viannia) naiffi* en GenBank, una especie previamente desconocida en Panamá, pero reconocida como un tipo de leishmaniasis cutánea transmitida ampliamente a través del norte y centro de Suramérica. Cepas distintas de la bacteria intracelular, *Wolbachia*, fueron detectadas en tres de veinte especies de mosquitos flebótomos, incluyendo *Lu. trapidoi*, en el cual frecuentemente concurrió con la presencia de *Leishmania*.

**Conclusiones**

Métodos morfológicos y moleculares se usaron para examinar un ensamblaje de veinte especies de mosquitos flebótomos que ocurren en los bosques del área del Canal de Panamá. Dos de esas especies, miembros de clados distintos, resultaron ser portadores de *Leishmania* a altas frecuencias por lo cual son transmisores probables de leishmaniasis a los humanos y otros mamíferos. Una sola especie de *Leishmania*, identificada con alta confianza como *Le. naiffi* es portada por ambas especies. Se sabe que *Le. naiffi* es causante de leishmaniasis cutánea in Suramérica pero hasta ahora nunca ha sido reportada o implicada en casos en Panama, y implica la posibilidad de que su rango se ha expandido recientemente a incluir el istmo o que ocurre debido a una introducción reciente. La coocurrencia de *Leishmania* y *Wolbachia* en *Lu.trapidoi* identifica un transmisor importante de la enfermedad como objetivo potencial para introducción de genes usando una invasión de población de *Wolbachia*.

**Palabras clave:** *Lutzomyia*, *Leishmania naiffi*, Panamá, código de barras de ADN, CO1, 18S, ITS-1, filogenia, *Wolbachia*, MLST, Psychodidae, Phlebotominae.
